# Effective in-service training design and delivery: evidence from an integrative literature review

**DOI:** 10.1186/1478-4491-11-51

**Published:** 2013-10-01

**Authors:** Julia Bluestone, Peter Johnson, Judith Fullerton, Catherine Carr, Jessica Alderman, James BonTempo

**Affiliations:** 1 Jhpiego Corporation, 1615 Thames Street, Baltimore, MD 21231, USA; 2 Independent Consultant, San Diego, CA, USA; 3 Research Assistant, Baltimore, MD, USA

**Keywords:** In-service training, Continuing professional education, Continuing medical education, Continuing professional development

## Abstract

**Background:**

In-service training represents a significant financial investment for supporting continued competence of the health care workforce. An integrative review of the education and training literature was conducted to identify effective training approaches for health worker continuing professional education (CPE) and what evidence exists of outcomes derived from CPE.

**Methods:**

A literature review was conducted from multiple databases including PubMed, the Cochrane Library and Cumulative Index to Nursing and Allied Health Literature (CINAHL) between May and June 2011. The initial review of titles and abstracts produced 244 results. Articles selected for analysis after two quality reviews consisted of systematic reviews, randomized controlled trials (RCTs) and programme evaluations published in peer-reviewed journals from 2000 to 2011 in the English language. The articles analysed included 37 systematic reviews and 32 RCTs. The research questions focused on the evidence supporting educational techniques, frequency, setting and media used to deliver instruction for continuing health professional education.

**Results:**

The evidence suggests the use of multiple techniques that allow for interaction and enable learners to process and apply information. Case-based learning, clinical simulations, practice and feedback are identified as effective educational techniques. Didactic techniques that involve passive instruction, such as reading or lecture, have been found to have little or no impact on learning outcomes. Repetitive interventions, rather than single interventions, were shown to be superior for learning outcomes. Settings similar to the workplace improved skill acquisition and performance. Computer-based learning can be equally or more effective than live instruction and more cost efficient if effective techniques are used. Effective techniques can lead to improvements in knowledge and skill outcomes and clinical practice behaviours, but there is less evidence directly linking CPE to improved clinical outcomes. Very limited quality data are available from low- to middle-income countries.

**Conclusions:**

Educational techniques are critical to learning outcomes. Targeted, repetitive interventions can result in better learning outcomes. Setting should be selected to support relevant and realistic practice and increase efficiency. Media should be selected based on the potential to support effective educational techniques and efficiency of instruction. CPE can lead to improved learning outcomes if effective techniques are used. Limited data indicate that there may also be an effect on improving clinical practice behaviours. The research agenda calls for well-constructed evaluations of culturally appropriate combinations of technique, setting, frequency and media, developed for and tested among all levels of health workers in low- and middle-income countries.

## Background

The need to increase the effectiveness and efficiency of both pre-service education and continuing professional education (CPE) (in-service training) for the health workforce has never been greater. Decreasing global resources and a pervasive critical shortage of skilled health workers are paralleled by an explosion in the increase of and access to information. Universities and educational institutions are rapidly integrating different approaches for learning that move beyond the classroom [1]. The opportunities exist both in initial health professional education and CPE to expand education and training approaches beyond classroom-based settings.

An integrative review was designed to identify and review the evidence addressing best practices in the design and delivery of in-service training interventions. The use of an integrative review expands the variety of research designs that can be incorporated within a review’s inclusion criteria and allows the incorporation of both qualitative and quantitative information [2]. Five questions were formulated based on a conceptual model of CPE developed by the Johns Hopkins University Evidence-Based Practice Center (JHU EPC) for an earlier systematic review of continuing medical education (CME) [3]. We asked whether: 1. particular educational techniques, 2. frequency of instruction (single or repetitive), 3. setting where instruction occurs, or 4. media used to deliver the instruction make a difference in learning outcomes; and, 5. if there was any evidence regarding the desired outcomes, such as improvements in knowledge, skills or changes in clinical practice behaviours, which could be derived from CPE, using any mixture of technique, media or frequency.

## Methods

### Inclusion/exclusion criteria

Articles were included in this review if they addressed any type of health worker pre-service or CPE event, and included an analysis of the short-term evaluation and/or assessment of the longer-term outcomes of the training. We included only those articles published in English language literature. These criteria gave priority to articles that used higher-order research methods, specifically meta-analyses or systematic reviews and evaluations that employed experimental designs. Articles excluded from analysis were observational studies, qualitative studies, editorial commentary, letters and book chapters.

### Search strategy

A research assistant searched the electronic, peer-reviewed literature between May and June 2011. The search was conducted on studies published in the English language from 2000 to 2011. Multiple databases including PubMed, the Cochrane Library and Cumulative Index to Nursing and Allied Health Literature (CINAHL) were utilized in the search. Medical subject headings (MeSH) and key search terms are presented below in Table 1.

**Table 1:** Medical subject headings (MeSH) and key search terms

		
Group-based education	Asynchronous distance learning	Nursing education
Facility-based education	Synchronous distance learning	Medical education
On-the-job education	Online learning	Teaching methods
Group-based training	Distance learning	Health care professionals
Facility-based training	Continuing medical education	Education methods
On-the-job training	Continuing nursing education	Continuing education methods
Point-of-care training		Nursing education methods
Mobile technologies		Medical education methods

### Study type, quality assessment and grade

An initial review of titles and abstracts produced 244 results. We identified the strongest studies available, using a range of criteria tailored to the review methodology. Initial selection criteria were developed by a panel of experts. Grading and inclusion criteria are presented in Table 2. The grading criteria were adapted from the Oxford Centre for Evidence-Based Medicine (OCEMB) levels of evidence model [4]. Grading of studies included within systematic reviews was reported by authors of those reviews and was not further assessed in this integrative review. Therefore, reference to quality of studies in our report refers to those a priori judgments. Only tier 1 articles (grades 1 and 2) were included in our analysis.

**Table 2:** Grading criteria

**Design**	**Type of groups**	**Literature grade**	**Tier**
Meta-analysis or systematic review	NA	1	
Experimental	Between subjects (experimental and control)	2	1
	Within subjects (crossover)	2	
Quasi-experimental	Non-equivalent control group	3	
	Repeated measures	3	
Pre-experimental	Comparison group	4	
	Pre-test/post-test	4	2
	Post-test only	5	

*NA* not applicable.

**Figure 1:**
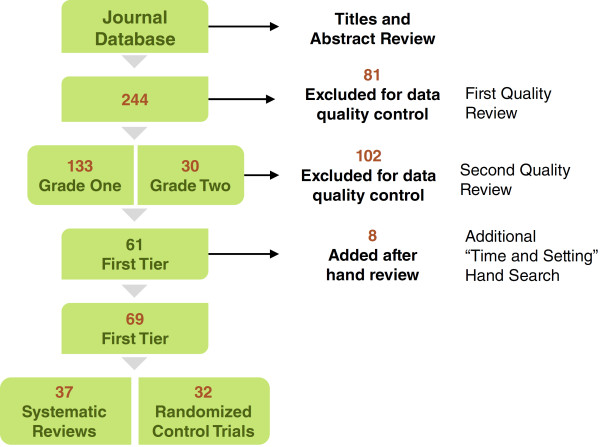
Inclusion process for articles included in the analysis.

After prioritization of the articles, 163 tier 1 articles were assessed by a senior public health professional to determine topical relevance, study type and grade. A total of 61 tier 1 studies were selected to be included in the analysis following this second review. An additional hand search of the reference lists cited in published studies was conducted for topics that were underrepresented, specifically on the frequency and setting of educational activities. This search added eight articles for a total of 69 studies, including 37 systematic reviews and 32 randomized controlled trials (RCTs), see inclusion process for articles included in analysis, Figure 1.

**Figure 2:**
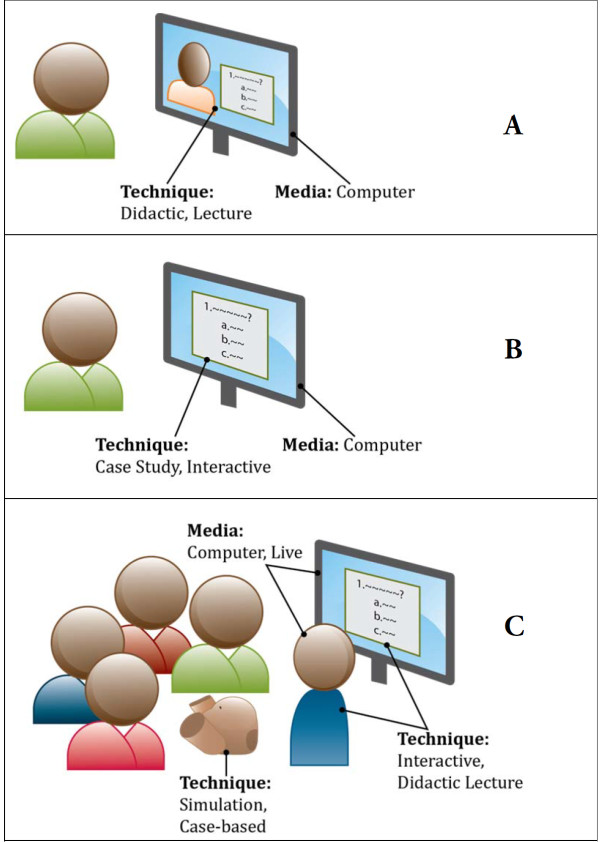
Illustration of categorization terminology in panels a-c.

A data extraction spreadsheet was developed, following the model offered in the Best Evidence in Medical Education (BEME) group series [5] and the conceptual model and definition of terms offered by Marinopoulos et al. in the JHU EPC earlier review of CME [3]. Categorization decisions were necessary in cases when the use of terminology was inconsistent with the Marinopoulos et al. definitions of terms for CPE [3]. For example, an article that analysed 'distance learning’ as a technique and used the computer as the medium to deliver an interactive e-learning course was coded and categorized as an 'interactive’ technique delivered via 'computer’ as the medium of instruction. See illustration of categorization terminology in panels A, B, and C, Figure 2, for an illustration of how terminology was used to categorize and organize articles for analysis.

## Results

Selected articles that best represent common findings and outcomes (effects) of CPE are discussed in the results and discussion sections; the related tables present all the articles analysed and categorized for that topic, and each article is included only once. Relevant information obtained from educational psychology literature is referenced in the discussion.

### Techniques

The articles or studies that specifically addressed educational techniques are summarized in Table 3. Technique refers to the educational methods used in the instruction. Technique descriptions are based on the Marinopoulos et al. definitions of terms [6] and reflect the approaches defined in the articles analysed.

**Table 3:** Summary of articles focused on techniques

**Citation**	**Study design**	**Participants**	**Intervention**	**Key findings**
Aki E et al. 2010	Systematic review: five articles reviewed to determine the effectiveness of educational gaming on learning	Mostly medical students	Technique: educational games	Findings in three of the five RCTs suggested but did not confirm a positive effect of the games on medical students’ knowledge.
			Media: multiple	
			Frequency: NR	
Blaya J et al. 2010	Systematic review: 45 articles included for review, only three related to POC support, included qualitative and quantitative data	Nurses in developing countries	Technique: didactic vs POC	POC findings: studies were weak but indicated knowledge improved and increased rapport in trusting personal judgment.
			Media: computer-based vs live	
			Frequency: NR	
Bruppacher H et al. 2010	Prospective, single-blinded RCT to determine if simulation or interactive techniques are better for teaching weaning a patient from anaesthesia	Anaesthesiology trainees, post-graduate year 4	Technique: simulation vs interactive	The simulation group scored significantly higher than the seminar group at both post-test and retention test. Clinical decision-making/psychomotor skills can be acquired via simulation.
		I = 10, C = 10	Media: live	
		Country: China	Frequency: single	
			Intervention group received simulation-based training; control group received an interactive seminar.	
Daniels K et al. 2010	Prospective RCT to determine if simulation is more effective than didactic in obstetric emergency management	Residents and labour and delivery nurses	Technique: simulation vs interactive	Simulation-trained teams had superior performance scores when tested in a labour and delivery drill. In an academic training programme, didactic and simulation-trained groups showed equal results on written test scores.
		I = 16, C = 16	Media: live	
		Country: USA	Frequency: single	
			Intervention group received simulation-based training; control group received an interactive seminar.	
De Lorenzo R and Abbott C 2004	RCT to determine if the adult learning model improves student learning in terms of cognitive performance and perception of proficiency in military medic training	Army medic students	Technique: interactive vs didactic	The adult learning model offered only a modest improvement in cognitive evaluation scores over traditional teaching. Additionally, students in the traditional teaching model assessed themselves as proficient more frequently than instructors, whereas instructor and student perception of proficiency were more closely matched in the adult learning model.
		n = 150, I = 81, C = 69	Media: live	
		Country: USA	Frequency: single	
			Intervention group emphasized the principles of adult learning including small group interactive approach, self-directed study, multimedia didactics and intensive integrated practice of psychomotor skills; control group received a traditional, lecture-based course.	
Harder BN 2010	Systematic review: 23 articles reviewed to evaluate the use of clinical simulation in health care education	Health professionals	Technique: simulation	Inconclusive evidence about the use of simulation due to a low number of studies. However, the use of simulation, as opposed to other education and training methods (motor skills laboratory sessions with task trainers, computer-based instruction and lecture classes), increased students’ clinical skills in the majority of studies.
			Media: multiple	
			Frequency: single	
Herbert C et al. 2004	RCT to assess the impact of individualized feedback and live, interactive group education on prescriptive practices	Physicians	Technique: audit and feedback vs interactive plus audit and feedback vs interactive session only vs nothing	Increase in prescribing preference for correct drug class in module and “prescribing portraits” (graphic comparisons between individual, group and evidence based prescribing practices) group. Evidence-based educational interventions combining personalized prescribing feedback with interactive group discussion can lead to modest but meaningful changes in physician prescribing.
		I1 = 48, audit and feedback only; I2 = 47, interactive module only; I3 = 49, interactive plus audit and feedback; C = 56, nothing	Media: live	
		4,394 charts reviewed	Frequency: single	
		Country: Canada		
Issenberg S et al. 2005	Systematic review: 109 studies reviewed to determine the use of high-fidelity medical simulations that lead to most effective learning	Health professionals	Technique: simulation	The weight of the best available evidence suggests that high-fidelity medical simulations facilitate learning under the right conditions. These conditions include: providing feedback, repetitive practice, curriculum integration, range of difficulty, multiple learning strategies, capture clinical variation, controlled environment, individualized learning, defined outcomes and simulator validity.
			Media: multiple	
			Frequency: both single and multiple	
Lamb D 2007	Literature review: nine articles reviewed to determine effectiveness of experiential (focused on simulations) learning	Health professionals	Technique: simulation	None of the studies showed conclusively that simulated learning improves patient outcome; however, evidence suggests human patient simulators to be advantageous over other modalities. They have been proven to be at least as effective as traditional teaching by didactic methods. Both human patient simulators (models) and computer-simulations may be effective.
			Media: multiple	
			Frequency: both single and multiple	
Laprise R et al. 2009	Cluster randomized trial of 122 general practitioners to determine if chart audits and feedback reminders after a CME event lead to better adherence to clinical guidelines	General practitioners	Technique: audit and feedback plus interactive vs interactive only	This study demonstrated significantly improved adherence in the intervention group using chart audits vs CME alone. The magnitude of the difference observed between the two groups in absolute pre-post intervention change is consistent with previous studies on the effectiveness of chart prompting in preventive care.
		n = 122, I = 61, C = 61	Media: live	
		Chart audit of 2,344 consenting patient charts	Frequency: single vs multiple	
		Country: Canada	Intervention group and control group received the same CME intervention, a 2-hour live, interactive workshop. The intervention group also received six monthly follow-up visits from a nurse that included chart screening, audits and feedback, and a print-based checklist distribution and print summary of expert recommendations.	
Lin C et al. 2010	RCT to determine if peer-tutored, PBL is preferable to didactic-based instruction for teaching nursing ethics	Nursing students	Technique: PBL vs didactic	Peer-tutored, PBL was shown to be more effective than conventional lecture-type teaching. Peer-tutored, PBL has the potential to enhance the efficacy of teaching nursing ethics in situations in which there are personnel and resource constraints.
		I = 72, C = 70	Media: live	
		Country: Taiwan	Frequency: single	
			Intervention group received PBL technique; control group received didactic-based instruction.	
McGaghie W et al. 2009^a^	Systematic review: nine of the JHU EPC systematic review articles reviewed to determine the effectiveness of simulation methods in medical education outside of CME	Health professionals	Technique: simulation	Due to a low number of studies, evidence on simulation methods is inconclusive. However, the direction of evidence points to the effectiveness of simulation training, especially for psychomotor and communication skills. Data analysis revealed a highly significant 'dose-response’ relationship among practice and achievement, with more practice producing higher outcome gains.
			Media: multiple	
			Frequency: both single and multiple	
Merien A et al. 2010	Systematic review: eight articles reviewed to determine the effectiveness of team-based training for obstetric care	Health professionals	Technique: team-based	Due to a low number of studies, evidence on teamwork training in simulation is inconclusive. However, introduction of multidisciplinary teamwork training with integrated acute obstetric training interventions in a simulation setting is potentially effective in the prevention of errors, thus improving patient safety in acute obstetric emergencies.
			Media: live	
			Frequency: NR	
Murad MH et al. 2010	Systematic review: 59 articles (enrolled 8,011 learners) reviewed to determine effectiveness of self-directed learning	Health professionals	Technique: self-directed	Moderate-quality evidence suggests that self-directed learning in health professions education is associated with moderate improvement in the knowledge domain compared with traditional teaching methods, and may be as effective in the skills and attitudes domains.
			Media: multiple	
			Frequency: NR	
Perry M et al. 2011	Systematic review: six articles representing five studies were reviewed to determine the effect of educational interventions in primary dementia care	Health professionals	Technique: multiple	Interactive workshops and decision support systems led to increased detection rates. Evidence shows moderate improvements in knowledge and techniques that required active participation tended to improve detection rates.
			Media: multiple	
			Frequency: both single and multiple	
Reynolds A et al. 2010	RCT to compare students’ knowledge using either simulation or didactic lecture	Midwifery students	Technique: simulation vs didactic	A significantly higher short-term reinforcement of knowledge and greater learner satisfaction was obtained using simulation-based training compared to image-based lectures when teaching routine management of normal delivery and resolution of shoulder dystocia to midwives in training.
		I = 26, C = 24	Media: live	
		Country: Portugal	Frequency: single	
			Intervention group received simulation-based training; control group received didactic lectures with print visuals.	
Smits P et al. 2003	RCT to compare effectiveness of PBL vs didactic for management of mental health problems	Post-graduate medical trainees	Technique: PBL vs didactic	The study found that both PBL and didactic-based instruction were effective, but had no statistical difference. The PBL programme appeared to be more effective than the lecture-based programme in improving performance, but received less favourable evaluations.
		I = 59, C = 59	Media: live	
		Country: the Netherlands	Frequency: single	
			Intervention group received PBL technique; control group received didactic-based instruction.	
Steadman R et al. 2006	RCT to determine if simulation is better than PBL for teaching assessment and management skills	4th year medical students	Technique: simulation vs PBL	Simulation-based teaching was superior to PBL for the acquisition of critical assessment and management skills.
		I = 15, C = 16	Media: live	
		Country: USA	Frequency: single	
			Intervention group received simulation-based teaching; control group received PBL.	
Sturm L et al. 2008	Systematic review: 11 articles reviewed to determine if skills acquired by simulation-based training transfer to the operative setting	Surgeons	Technique: simulation	Due to limited quality and methodology and a lack of relevant studies, a weak conclusion can be made supporting the transfer of skills developed in simulation to the operative setting. Evidence from one study showed better performance for participants who received simulation-based training before undergoing patient-based assessment than their counterparts who did not receive previous simulation training.
			Media: multiple	
			Frequency: both single and multiple	
Werb S and Matear D 2004	Systematic review: three systematic reviews and nine original research articles reviewed to examine evidence-based clinical teaching and faculty continuing education	Allied health professionals	Technique: PBL	PBL and evidence-based health care interventions were effective in increasing students’ knowledge of medical topics and their ability to search, evaluate and appraise medical literature. Dental students in a PBL curriculum, emphasizing evidence-based practices, scored higher on the National Dentistry Boards, Part I, than students in traditional curricula.
			Media: multiple	
			Frequency: both single and multiple	
White M et al. 2004	RCT to investigate effectiveness of PBL vs didactic for asthma management	Physicians	Technique: PBL vs didactic	There was no significant difference in knowledge gained or satisfaction with the facilitator between the PBL group and the lecture-based group. The PBL group rated the educational value higher than the didactic group.
		I = 23, C = 29	Media: live	
		Country: Canada	Frequency: single	
			Intervention group received PBL technique; control group received didactic-based instruction.	
Young J and Ward J 2002	Randomized trial to determine the effect of self-directed (distance) learning on knowledge, attitudes and practices related to smoking cessation	Family physicians	Technique: self-directed vs reading	Modest changes from baseline to post-test for both the distance learning group and self-directed group suggest a lack of significant evidence to support a distance or self-directed approach to address changes in practice.
		I = 26, C = 27	Media: print	
		Country: Australia	Frequency: single	
			Intervention group received a self-directed learning module; control group received guidelines only.	
Yuan H et al. 2008	Systematic review: 10 studies reviewed to determine the evidence to support PBL	Nursing students	Technique: PBL	Inconclusive evidence to support PBL. While several studies showed increased reported self-confidence in ability to make decisions, and several showed increased skills in critical thinking questions from the PBL group, overall findings were inconclusive due to a lack of quality studies.
			Media: multiple	
			Frequency: both single and multiple	
Zurovac D et al. 2011	Cluster RCT at 107 rural health facilities to determine if text-message reminders would improve provider adherence to national malaria treatment guidelines	Health professionals	Technique: reminders	The use of mobile technology showed significant improvement in case management practice for paediatric malaria cases among physicians with repetitive text-message reminders compared to control group.
		119 health workers	Media: mobile phone	
		Case-management practices were assessed for 2,269 children who needed treatment	Frequency: repetitive	
		I = 1,157, C = 1,112	Intervention group received repetitive text messages over a 6-month period; control group received nothing.	
		Country: Kenya		

^a^JHU EPC systematic review. *C* Control, *CME* Continuing medical education, *I* Intervention, *JHU EPC* Johns Hopkins University Evidence-Based Practice Center, *NR* Not reported, *PBL* problem-based learning, *POC* point-of-care, *RCT* randomized controlled trial.

#### ***Case-based: use of created or actual clinical cases that present materials and questions***

Though case-based learning was not specifically compared with other techniques in the literature reviewed, it was often noted as a method in articles that discussed interactive techniques. Case-based learning was also noted as a technique used for computer-delivered CPE courses. Triola et al. compared types of media utilized for case-based learning and found positive learning outcomes both with the use of a live standardized patient and a computer-based virtual patient [7].

#### ***Didactic/lecture: presenting knowledge content; facilitator determines content, organization and pace***

Lecture was often referred to in the literature as traditional instruction, lecture-based or didactic teaching. Didactic instruction was not found to be an effective educational technique compared with other methods. Two studies [8,9] found no statistical difference in learning outcomes, and three studies found didactic to be less effective than other techniques [10-12]. Reynolds et al. compared didactic instruction with simulation. The study was limited by small sample size (n = 50), but still demonstrated that the simulation group had a significantly higher mean post-test score (*P* <0.01) and overall higher learner satisfaction [12].

Several systemic reviews that compared didactic instruction to a wide variety of teaching approaches also identified didactic instruction as a less effective educational technique [13-15].

#### ***Feedback: providing information to the learner about performance***

Multiple articles identified feedback as important for outcomes [16-18]. Herbert et al. compared individualized feedback in the form of a graphic (a prescribing portrait based on personal history of drug-prescribing practices) to small group discussion of the same material and found that both the feedback and the live, interactive session were somewhat effective at changing physician’s prescribing behaviours [16]. The Issenberg et al. systematic review of simulation identified practice and feedback as key for effective skill development [17]. A Cochrane review of the evidence to support CPE suggested the importance of feedback and instructor interaction in improving learning outcomes [18].

#### ***Games: competitive game with preset rules***

The use of games as an instructional technology was addressed in one rigorous systematic review. The authors found only a limited number of studies, which were of low to moderate methodological quality and offered inconsistent results. Three of the five RCTs included in the review suggested that educational games could have a positive effect on increasing medical student knowledge and that they include interaction and allow for feedback [19].

#### ***Interactive: provide for interaction between the learner and facilitator***

Five articles specifically compared interactive CPE to other educational techniques. De Lorenzo and Abbot found interactive techniques to be moderately superior for knowledge outcomes than didactic lecture [10]. Two other studies found interactive techniques were more effective when feedback from chart audits was added to the intervention [16,20].

Three systematic reviews and one meta-analysis specifically noted the importance of learner interactivity or engagement in learning in achieving positive learning outcomes [21-24] (refer to summary of articles focused on outcomes).

#### ***Point-of-care (POC): information provided as needed, at the point of clinical care***

Two articles and one systematic review specifically addressed point-of-care (POC) as a technique. The systematic review included three studies and concluded that while the findings were weak, they did indicate that POC led to improved knowledge and confidence [25]. In an examination of media, Leung et al. determined that handheld devices were more effective than print-based, POC support, although outcome measures were self-reported behaviours [26]. You et al. found improved performance on a procedure among surgical residents who received POC mentoring via a video using a mobile device, compared with those who received only didactic instruction [27].

#### ***Problem-based learning (PBL): present a case, assign information-seeking tasks and answer questions about the case; can be facilitated or non-facilitated***

Four articles specifically compared problem-based learning (PBL) to other methods. One study identified PBL as slightly better [11], and two studies indicated it to be relatively equal to didactic instruction [8,9]. A systematic review of 10 studies on PBL reported inconclusive evidence to support the approach, although several studies reported increased critical thinking skills and confidence in making decisions [28].

#### ***Reminders: provision of reminders***

The Zurovac et al. study conducted in Kenya found that using mobile devices for repetitive reminders resulted in significant improvement in health care provider’s case management of paediatric malaria, and these gains were retained over a 6-month period [29]. Intention-to-treat analysis showed that correct management improved by 23.7% (95% confidence interval (CI) 7.6 to 40.0, *P* <0.01) immediately after intervention and by 24.5% (95% CI 8.1 to 41.0, *P* <0.01) 6 months later, compared with the control group [29]. Reminders were also noted as an effective technique by two of the systematic reviews [13,14].

#### ***Self-directed: completed independently by the learner based on learning needs***

This term was difficult to extract for analysis due to widely varying terminology. Some authors used the term 'distance learning’, and some used it to define the medium of delivery, rather than technique. This analysis specifically discusses articles that were consistent with the description for self-directed learning, even if the authors used different terminology.

A recent systematic review identified that moderate-quality evidence suggests a slight increase in knowledge domain compared with traditional teaching, but notes that this may be due to the increased exposure to content [30]. One RCT found modest improvements in knowledge using a self-directed approach, but noted it was less effective at impacting attitudes or readiness to change [31].

Multiple studies focused on use of the computer as the medium to deliver instruction and noted that self-directed instruction was equally (or more) effective as instructor-led didactic or interactive instruction and potentially more efficient.

Simulation may include models, devices, standardized patients, virtual environments, social or clinical situations that simulate problems, events or conditions experienced in professional encounters [17]. Simulation was noted as an effective technique for promotion of learning outcomes across the systematic reviews, particularly for the development of psychomotor and clinical decision-making skills. The systematic reviews all highlighted inconclusive and weak methodology in the studies reviewed, but noted sufficient evidence existed to support simulation as useful for psychomotor and communication skill development [32-34] and to facilitate learning [35]. The systematic review by Lamb suggests that patient simulators, whether computer or anatomic models, are one of the more effective forms of simulations [36].

Outcomes of the four separate RCTs indicated simulation was better than the techniques to which they were compared, including interactive [37,38], didactic [12] and problem-based approaches [35]. A study by Daniels et al. found that although knowledge outcomes were similar between the interactive and simulation groups, the simulation team performance in a labour and delivery clinical drill was significantly higher for both shoulder dystocia (11.75 versus 6.88, *P* <0.01) and eclampsia (13.25 versus 11.38, *P* = 0.032) at 1 month post-intervention [38].

Simulation was also found to be useful for identifying additional learning gaps, such as a drill on the task of mixing magnesium sulfate for administration [39]. A systematic review focused on resuscitation training identified simulation as an effective technique, regardless of media or setting used to deliver it [40].

#### ***Team-based: providing interventions for teams that provide care together***

Articles discussed here focused on the technique of providing training to co-workers engaged as learning teams. One systematic review of eight studies found that there is limited and inconclusive evidence to support team-based training [41]. Two of the articles reporting on the same CPE study did not identify any improvements in performance or knowledge acquisition with the addition of using a team-based approach [39,42].

### Frequency

This review included consideration of frequency, comparing single versus repetitive exposure. The findings regarding frequency are summarized in Table 4.

**Table 4:** Summary of articles focused on frequency

**Citation**	**Study design**	**Participants**	**Intervention**	**Key findings**
Kerfoot BP et al. 2007	RCT to determine if spacing principles can improve acquisition and retention of medical knowledge	Five cohorts with 76 to 80 urology residents in each cohort	Frequency: multiple vs single	Conclusive evidence to support repetitive, spaced education in online learning, since residents in the spaced education cohort demonstrated significantly greater online test scores than those in the bolus cohort. The scores for the spaced cohort remained stable with no overtime, while test scores in the bolus cohort demonstrated a significant linear decrease.
		Of 537 participants, 400 (74%) completed the online staggered tests and 515 (96%) completed the In-Service Examination	Technique: self-directed	
		Cohort 1 = bolus, single intervention; Cohort 2 = multiple, spaced intervention	Media: Internet-based	
		Country: USA and Canada	Cohort 1 received bolus education of 96 study questions (June 2005); Cohort 2 received daily emails over 27 weeks (June to December 2005), each with one to two questions in spaced pattern. In November 2005, all participants completed the urology exam. Participants were randomized to five cohorts and completed a 32-item online test at staggered time points (1 to 14 weeks) after completion of Spaced Education.	
Kerfoot BP et al. 2009	RCT to determine if Spaced Education is an effective form of CME	Urologists and urology residents	Frequency: multiple vs single	Conclusive evidence to support the use of ISE programmes. Knowledge scores of ISE intervention were statistically significantly higher than those of the control bolus method.
		Completed by 71% of urologists and 83% of residents	Technique: self-directed	
		Cohort 1 = 80 urologists, 160 residents, completed by 196; Cohort 2 = 80 urologists, 160 residents, completed by 182	Media: Internet-based	
		Country: USA (March to July 2007)	A total of 160 urologists and 320 urology residents were randomized to one of two cohorts. Participants were stratified by training level (urologist in practice vs resident) and urology training year (residents only) and were block randomized (block size = 8) to one of two cohorts. Participants in Cohort 1 received the 3-cycle ISE course on the HP CPGs, with 24 control items on the SIA CPGs in cycle 3. Participants in Cohort 2 received the 3-cycle ISE course on SIA CPGs, with 24 control items on HP CPGs in cycle 3. The trial was structured in this manner to allow the topic-specific learning gains from the ISE courses to be identified in cycle 3. Since the 24 items are presented simultaneously to both cohorts in cycle 3, the learning gains of physicians who had completed two cycles of the ISE programme could be directly compared with those physicians who were presented with the material for the first time (controls).	
Kerfoot BP et al. 2010	RCT to determine if Spaced Education can effect knowledge transfer and the ability to make diagnostic decisions	Urology residents	Frequency: multiple vs single	Conclusive evidence to support spaced, web-based education compared to WBT. Spaced education demonstrated a statically significant increase in knowledge and long-term retention of knowledge compared with bolus web-based modules that delivered the same content of histopathology diagnostic skills.
		Cohort 1 = 164; Cohort 2 = 194	Technique: self-directed	
		Country: USA (June 2007 to June 2008)	Media: Internet-based	
			Transfer and retention of diagnostic skills between Spaced Education vs bolus, WBT	
			All residents were sent both spaced education and WBT, but the set of topics delivered by each method varied by cohort. Residents in Cohort 1 received three cycles of spaced education on prostate-testis (weeks 1 to 4, 5 to 8, and 13 to 16) and three WBT modules on bladder-kidney (weeks 14 to 16). Residents in Cohort 2 received three cycles of spaced education on bladder-kidney (weeks 1 to 4, 5 to 8, and 13 to 16) and three WBT modules on prostate-testis (weeks 14 to 16). The spaced education items were delivered each weekday through emails containing one question/answer, and the spaced education material was distributed in three cycles or repetitions to take advantage of the spacing effect. The WBT used the identical content and delivery system, with the questions aggregated into three 20-question modules delivered through separate emails in week 14. The trial was specifically structured to ensure that within a given set of topics (bladder-kidney or prostate-testis) the only difference between intervention cohorts was the spacing of content.	

*CME* Continuing medical education, *CPG* Clinical practice guideline, *HP* Haematuria and priapism, *ISE* Interactive spaced education, *RCT* Randomized controlled trial, *SIA* Staghorn calculi, infertility, and antibiotic use, *WBT* Web-based teaching.

The three articles focused on frequency all support the use of repetitive interventions. These studies evaluated repetition using the Spaced Education platform (now called Qstream), an Internet-based medium that uses repeated questions and targeted feedback. The evidence from these three articles demonstrated that repetitive, time-spaced education exposures resulted in better knowledge outcomes, better retention and better clinical decisions compared with single interventions and live instruction [43-45].

The use of repetitive or multiple exposures is supported in other systematic reviews of the literature, as well as one RCT conducted in Kenya that used repeated text reminders and resulted in a significant improvement in adherence to malaria treatment protocols [29].

### Setting

Setting is the physical location within which the instruction occurs. We identified three articles that looked specifically at the training setting. The findings regarding setting are summarized in Table 5. Two of them stemmed from the same intervention. Crofts et al. specifically addressed the impact of setting and technique (team-based training) on knowledge acquisition and found no significant difference in the post-score based on the setting [42]. A systematic review of eight articles evaluating the effectiveness of team-based training for obstetric care did not find significant differences in learning outcomes between a simulation centre and a clinical setting [41].

**Table 5:** Summary of articles focused on setting

**Citation**	**Study design**	**Participants**	**Intervention**	**Key findings**
Coomarasamy A and Khan K 2004 (link to the follow-up study, Raza A et al. 2009)	Systematic review: 23 articles reviewed to determine the effect of stand-alone compared to clinically integrated teaching in EBM	Post-graduate physicians, allied health professionals	Technique: multiple, focus on case-based	Sufficient evidence to support the use of clinically integrated teaching over stand-alone education. While stand-alone teaching improved knowledge, there were no improvements in skills, attitudes or behaviours, whereas clinically integrated teaching showed improvements in knowledge, skills, attitude and behaviour.
			Media: live	
			Frequency: both single and multiple	
Crofts J et al. 2007	Prospective RCT to explore if knowledge acquisition is influenced by training setting or teamwork training	Senior doctors, junior and senior midwives	Technique: team-based vs interactive	Statistical evidence supported the use of live, multi-professional, obstetric emergency training to increase midwives’ and doctors’ knowledge of obstetric emergency management. However, neither the location of training either in a simulation centre or in local hospitals, nor the inclusion of teamwork training, made any significant difference to the acquisition of knowledge in obstetric emergencies.
		Total of 140 participants; interdisciplinary teams of four or six in four blocks	Media: live	
		Country: UK	Frequency: single	
			I1 = 1-day interactive at hospital (no team-based training); I2 = 1-day interactive at simulation centre (no team-based training); I3 = 2-day team training at hospital; I4 = 2-day interactive in simulation centre	
			Main outcome measured by a 185 multiple-choice questionnaire completed 3 weeks before and 3 weeks after the training intervention.	
Ellis D et al. 2008	Same study design as Crofts et al. 2007	Same participants as Crofts et al. 2007	Same intervention as Crofts et al. 2007	Statistical evidence to support the use of live, eclampsia training to increase providers’ performance rate for completion of basic tasks. Neither the location (simulation centre or in local hospitals), nor the inclusion of teamwork training made any significant difference to the performance results for basic task completion.

*EBM* Evidence-based medicine, *I* Intervention.

Coomarasamy and Khan conducted a systematic review and compared classroom or stand-alone versus clinically integrated teaching for evidence-based medicine (EBM). Their review identified that classroom teaching improved knowledge, but not skills, attitudes or behaviour outcomes; whereas clinically integrated teaching improved all outcomes [46]. This finding was supported by the Hamilton systematic review of CPE, which suggests that teaching in a clinical setting or simulation setting is more effective (Table 1), as well as the Raza et al. systematic review of 23 studies to evaluate stand-alone versus clinically integrated teaching. This review suggested that clinically integrated teaching improved skills, attitudes and behaviour, not just knowledge [18].

### Media

Media refers to the means used to deliver the curriculum. The majority of RCTs compared self-paced or individual instruction delivered via computer versus live, group-based instruction. The findings regarding media are summarized in Table 6.

**Table 6:** Summary of articles focused on media used to deliver instruction

**Citation**	**Study design**	**Participants**	**Intervention**	**Key findings**
Augestad K and Lindsetmo R 2009	Systematic review: 51 articles reviewed to determine usefulness of videoconferencing as a clinical and educational tool	Surgeons	Media: video	Review discussed primarily observational data on the use of videoconferencing for provision of lecture, mentoring and POC support for emergencies or trauma settings. Methodology of studies is weak, but shows promise for providing POC and mentoring to rural settings from specialists in other geographical areas.
		Country: Norway and developed countries	Technique: multiple	
			Frequency: NR	
Bloomfield J et al. 2010	RCT to test if the theory and skill of handwashing can be taught more effectively when taught using computer-assisted learning compared to conventional face-to-face teaching	Nursing students	Media: computer-based vs live	The computer-assisted learning module was an effective strategy for teaching both theory and practice of handwashing to nursing students and was found to be at least as effective as conventional, face-to-face teaching methods. However, this finding must be interpreted with caution in light of sample size and attrition rates.
		n = 242, I = 113, C = 118	Techniques: multiple	
		Country: UK	Frequency: single	
			Intervention group received theory via computer-based module; control group via instructor-led. The objectives and content were the same, both groups included practice opportunities.	
Bradley P et al. 2005	Prospective RCT and qualitative evaluation to compare self-directed, computer-based learning to traditional, live, interactive education techniques	Medical students	Technique: self-directed vs interactive	There were no differences in outcomes for the computer-based group compared to the live, interactive group in knowledge acquisition, critical appraisal skills or attitudes toward EBM. This trial and its accompanying qualitative evaluation suggest that self-directed, computer-assisted learning may be an alternative format for teaching EBM.
		I = 85, C = 90		
		Country: Norway	Media: computer-based vs live	
			Frequency: single	
			Intervention group received self-directed, computer-based modules on EBM; control group received live, interactive sessions.	
Choa et al. 2008	Single-blinded, cluster randomized trial to compare the effectiveness of audiovisual animated CPR instruction with audio, dispatcher-assisted instruction in participants with no previous CPR training; both via mobile phones	Allied health professionals, hospital employees	Media: mobile, audiovisual animation vs audio instructions from live dispatcher	Audiovisual animated CPR instruction via mobile phone resulted in better scores in checklist assessment and time interval compliance in participants without CPR skill compared to those who received CPR instructions from a dispatcher. However, the accuracy of important psychomotor skill measures was unsatisfactory in both groups.
			Technique: POC	
		I = 44, C = 41		
		Country: Korea		
			Frequency: single	
			Intervention group used mobile phone application with audiovisual animation instructions for CPR; control group received audio guidance from a live dispatcher.	
Chui S et al. 2009	Experimental research design with two groups, one pre-test and two post-tests, to determine the effectiveness of computer-based interactive instruction vs video didactic instruction	Nurses	Media: computer-based vs video	Interactive, computer-assisted instruction increased student assessment correctness compared to video didactic instruction for in-service neurological nursing education after statistical adjustments for length of experience.
		I = 44, C = 40	Technique: self-directed interactive vs didactic	
		Country: Taiwan	Frequency: single	
			Intervention group received computer-based, interactive educational module; control group watched a video of a lecture.	
Curran V and Fleet L 2005	Systematic review to evaluate the nature and characteristics of the web-based CME, based on Kirkpatrick levels of evaluation; 86 studies were identified, majority were descriptive	Physicians	Media: Internet	Inconclusive evidence to identify the most effective characteristics of web-based CME due to a lack of studies focusing on performance change. Findings suggest web-based CME is effective in enhancing knowledge and attitudes. Several studies suggest interactive CME that requires participant activity and the chance to practice skills can effect changes in practice behaviours.
			Technique: multiple	
			Frequency: both single and multiple	
Farmer A et al. 2008	Systematic review: 23 studies reviewed to determine the usefulness of print-based materials in practice behaviours or clinical practice outcomes	Health care professionals	Media: print	Insufficient information to support the effectiveness of print-based educational materials compared to other interventions. Print materials may have a beneficial effect on process outcomes compared to no intervention, but not on clinical practice outcomes.
			Technique: didactic	
			Frequency: single	
Fordis M et al. 2005	RCT to determine if Internet-based CME can produce changes comparable to those produced via live, small group, interactive CME with respect to physician knowledge and behaviours that have an impact on patient care	Physicians	Media: Internet-based vs live, interactive	Internet-based CME can produce objectively measured changes in behaviour as well as sustained gains in knowledge that are comparable or superior to those realized from an effective, live, group-based activity. The Internet-based intervention was associated with a significant increase in the percentage of high-risk patients treated with pharmacotherapeutics according to guidelines compared to the live, group-based control group.
		n = 97; I = 49, randomly assigned Internet-based over 2 weeks; C1 = 44, single, live, interactive session; C2 = 18, from same sites received nothing	Technique: self-directed vs interactive	
			Frequency: single	
			Intervention group received Internet-based modules over 2 weeks; one control group received a live, interactive session and the other control group received nothing.	
		Country: USA		
Hadley J et al. 2010	Cluster RCT to evaluate the educational effectiveness of a clinically integrated e-learning course for teaching basic EBM among post-graduate medical trainees compared to a traditional lecture-based course of equivalent content	Post-graduate medical trainees, interns	Media: Internet vs live	An e-learning course in EBM was as effective in improving knowledge as a standard lecture-based course. There was no statistically significant difference in knowledge of participants in the e-learning course compared to the lecture-based course. The benefits of an e-learning approach include standardization of teaching materials and it is a potential cost-effective alternative to standard, lecture-based teaching.
			Techniques: multiple	
			Frequency: single	
			Intervention group received clinical integrated, e-learning course on EBM; control group received live, didactic-based course.	
		Seven clusters of 237		
		I = 88, C = 72		
		Country: UK		
Harrington S and Walker B 2004	RCT to determine effectiveness of computer-based training compared with the traditional, instructor-led format	Nurses	Media: computer-based vs live	The computer-based group significantly outperformed the instructor-led group on the knowledge sub-test at post-test (gain of 28% vs 26%). Participants reported linked, computer-based learning and researchers noted potential for efficiencies and cost reduction.
		n = 1,294, I = 670, C = 624	Technique: didactic vs self-directed	
		Country: USA	Frequency: single	
			Intervention group received self-directed, computer-based instruction; control group received instructor-led, live instruction. Both groups had the same objectives and content.	
Horiuchi S et al. 2009	RCT compared web-based to live instruction	Nurses or midwives	Media: Internet vs live	No significant differences in knowledge were observed between the web-based and face-to-face group. However, the web-based instruction was rated as more flexible and affordable and had a lower drop-out rate than the face-to-face programme.
		n = 93; C = 45, web-based; I = 48, live	Techniques: multiple	
			Frequency: single	
			Intervention group received web-based instruction; control group received didactic live instruction.	
		Country: Japan		
Kemper K et al. 2006	National randomized 2 x 2 factorial trial	Health professionals	Media: Internet	There were statistically significant improvements in knowledge, confidence and communication scores after the course for each of the Internet–based delivery methods, with no significant differences in any of the three outcomes by delivery strategy. Outcomes were better for those who paid for continuing education credit.
		n = 1,267; completion rate = 62%; Group 1 = 318; Group 2 = 318; Group 3 = 318; Group 4 = 313	Technique: self-directed	
			Frequency: single	
			Group 1: four modules delivered weekly over 10 weeks by email (drip-push); Group 2: modules accessible on web site with four reminders weekly for 10 weeks (drip-pull); Group 3: 40 modules delivered within 4 days by email (bolus-push); and Group 4: 40 modules available on the Internet with one email informing participants of availability (bolus-pull).	
		Country: USA		
Leung G et al. 2003	RCT to compare the effectiveness of mobile, POC support vs print-based job aids	4th year medical students	Media: mobile vs print	Both the PDA and pocket card groups showed improvements in scores for personal application and current use of EBM. The PDA group showed slightly higher scores in all five outcomes, whereas those for the pocket card group were not appreciably different from the previous rotation.
			Technique: POC	
		n = 169; I = 54; C/pocket card = 55; C/nothing = 55		
			Frequency: single	
			Intervention group given PDA devices with clinical decision support tools; one control group was given a pocket card containing guidelines and the other control group received no intervention.	
		Country: China		
Liaw S et al. 2008	Cluster randomized trial to determine the effectiveness of locally adapted practice guidelines and education about paediatric asthma management, delivered to general practitioners using interactive, small group workshops	General practitioners	Media: live vs print only	Using interactive small group workshops to disseminate locally adapted guidelines was associated with improvement in general practitioners’ knowledge and confidence to manage asthma compared to receiving guidelines alone in the control arm, but did not change their self-reported provision of written action plans.
		n = 29, randomly assigned; I = 18, live, interactive plus guidelines; C/guidelines only = 18; C/nothing = 15	Technique: interactive vs reading	
		Country: Australia		
			Frequency: single	
			Intervention group received live, interactive sessions plus guidelines; control groups received guidelines only and no intervention.	
Rabol L et al. 2010	Systematic review: 18 studies reviewed to determine outcomes of live, classroom-based, multi-professional team training	Health professionals	Media: live	Although most studies had weak design methods, findings from the 18 studies concluded that team-based training led to positive participant evaluation, knowledge gain and behaviour change. However, the impact on clinical outcomes was limited.
			Technique: multiple	
			Frequency: single	
Sulaiman N et al. 2010	Same study design as **Liaw S et al. 2008 for CPE intervention**, but used questionnaires to determine any impact on completing written action plans or patient outcomes	411 patient surveys from patients of three arms utilized in Liaw, S., et al. 2008 at baseline; 341 at follow-up	See Liaw S et al. 2008	The interactive, small group workshops failed to translate into increased ownership of written action plans, improved control of asthma or improved quality of life, compared to receiving guidelines alone or control intervention.
		Country: Australia		
Triola M et al. 2006	RCT to compare effectiveness of virtual patients to live, standardized patients for improving clinical skills and knowledge	Health professionals	Media: virtual patient vs live patient	Improvements in diagnostic abilities were equivalent in groups who experienced cases either live or virtually. There was no subjective difference perceived by learners. Using virtual cases has the potential for cost efficiencies.
		I = 23, C = 32	Technique: case-based	
		Country: USA	Frequency: single	
			Intervention group received two live, standardized patient cases and two virtual patient cases; control group received four standardized patient cases.	
Turner M et al. 2006	Randomized, controlled, crossover trial to compare efficacy, student preference and cost of web-based, virtual patient vs live, standardized patient	2nd year medical students	Media: virtual patient vs live patient	There was no statistical difference in learning outcomes between the web-based and standardized patient; however, students preferred the standardized patient format. Start-up costs were comparable, but the ongoing costs of the web-based format were less expensive, suggesting that web-based teaching may be a viable strategy.
		I = 25, C = 24	Technique: case-based	
		Country: USA	Frequency: single	
			Intervention group received web-based instruction for one topic, then standardized patient for another topic. This was reversed for the second cohort, or control group, standardized patient first followed by web-based instruction.	
Wutoh R et al. 2004	Systematic review: 16 articles reviewed to determine the effect of Internet-based CME interventions on physician performance and health care outcomes	Physicians	Media: Internet	Results demonstrate that Internet-based CME are just as effective in imparting knowledge as traditional formats of CME. However, there is a lack of quality studies to conclude significant positive changes in practice behaviour and additional studies are needed.
			Technique: multiple	
			Frequency: both single and multiple	
You J et al. 2009	Prospective, randomized study to investigate usefulness of video via mobile device as an instruction tool	Surgical residents	Media: mobile videoconferencing/feedback	The overall success rate for performing needle thoracocentesis was significantly higher for the mobile phone video intervention compared to the control group without aided instruction. Participants also rated the mobile phone intervention with significantly higher scores for instrument difficulty and procedure satisfaction.
		I = 24, C = 25	Technique: live, interactive with and without mobile POC feedback using video	
		Country: South Korea		
			Frequency: single	
			Both intervention groups had a didactic session, performed a thoracentesis on a manikin while using video on a mobile phone and received feedback from a live instructor; control group did not receive any video-aided guidance.	

*C* Control, *CME* Continuing medical education, *CPR* Cardiopulmonary resuscitation, *EBM* Evidence-based medicine, *I* Intervention, *NR* Not reported, *PDA* Personal digital assistant.

*POC* Point-of-care, *RCT* Randomized controlled trial.

#### ***Live versus computer-based***

Live instruction was found to be somewhat effective at improving knowledge, but less so for changing clinical practice behaviours. When comparing live to computer-based instruction, a frequent finding was that computer-based instruction led to either equal or slightly better knowledge performance on post-tests than live instruction. One of the few to identify a significant difference in outcomes, Harrington and Walker found the computer-based group outperformed the instructor-led group on the knowledge post-test and that participants in the computer-based group, on average, spent less time completing the training than participants in the instructor-led group [47].

Systematic reviews indicate that the evidence supports the use of computer-delivered instruction for knowledge and attitudes; however, insufficient evidence exists to support its use in the attempt to change practice behaviours. The Raza Cochrane systematic review identified 16 randomized trials that evaluated the effectiveness of Internet-based education used to deliver CPE to practicing health care professionals. Six studies showed a positive change in participants’ knowledge, and three studies showed a change in practice in comparison with traditional formats [18]. One systematic review noted the importance of interactivity, independent of media, in achieving an impact on clinical practice behaviours [48].

#### ***Mobile***

One article assessed the use of animations against audio instructions in cardiopulmonary resuscitation (CPR) using a mobile phone and found the group that had audiovisual animations performed better than the group that received live instruction over the phone in performing CPR; however, neither group was able to perform the psychomotor skill correctly [49]. Leung et al. found providing POC decision support via a mobile device resulted in slightly better self-reporting on outcome measures compared with print-based job aids, but that both the print and mobile groups showed improvements in use of evidence-based decision-making [26].

#### ***Print***

The systematic review of print-based materials conducted by Farmer et al. did not find sufficient evidence to support the use of print media to change clinical practice behaviours [50]. A comparison of the use of print-based guidelines to a live, interactive workshop indicated that those who completed live instruction were slightly better able to identify patients at high risk of an asthma attack. However, neither intervention resulted in changed practice behaviours related to treatment plans [51].

Multiple systematic reviews caution against the use of print only media, concluding that live instruction is preferable to print only. Another consistent theme was support for the use of multimedia in CPE interventions.

### Outcomes

Outcomes are the consequences of a training intervention. This literature review focuses on changes in knowledge, attitudes, psychomotor, clinical decision-making or communication skills, and effects on practice behaviours and clinical outcomes. All of the articles that focused on outcomes were systematic reviews of the literature and are summarized in Table 7.

**Table 7:** **Summary of articles focused on outcomes**: **knowledge**, **attitudes**, **types of skills**, **practice behaviour**, **clinical practice outcomes**

**Citation**	**Study design**	**Participants**	**Intervention**	**Key findings**
Alvarez M and Agra Y 2006	Systematic review: 18 articles reviewed to determine educational interventions in palliative care and their impact on practice behaviours	Physicians and other allied health professionals	Practice behaviours	Due to a lack of quality studies, there are insufficient data to conclude about the impact of palliative care interventions on primary care physician practice performance. Although improvements in knowledge, some attitudes and provider satisfaction were demonstrated, there were no significant effects reported on practice behaviours. Didactic education alone was found to be ineffective. Interventions involving multiple techniques, reminders and feedback were found to be more effective at changing behaviours.
			Technique: multiple	
			Media: live	
			Frequency: both single and multiple	
Berkhof M et al. 2010	Systematic review: 12 systematic reviews reviewed to determine effective educational techniques to teach communication to physicians	Physicians	Communication skills	Sufficient evidence from 12 systematic reviews to recommend training programmes last at least 1 day, are learner-centred and focus on practicing skills. The best training strategies within the programmes included role-play, feedback and small group discussions. Training programmes should include active, practice-oriented strategies. Oral presentations on communication skills, modeling and written information should only be used as supportive strategies.
			Technique: multiple	
			Media: multiple	
			Frequency: both single and multiple	
Bloom B 2005	Systematic review: 26 articles (all systemic reviews or meta-analyses) reviewed to examine effectiveness of current CME tools and techniques in changing physician clinical practices and improving patient health outcomes	Physicians	Practice behaviours and clinical practice outcomes	Sufficient evidence to conclude that interactive techniques (audit/feedback, academic detailing/outreach, reminders) are the most effective CME methods impacting practice outcomes and behaviours, while clinical guidelines and opinion leaders are less effective. Didactic presentations and distributing printed information had little to no effect on physician practice.
			Technique: multiple	
			Media: multiple	
			Frequency: both single and multiple	
Bordage G et al. 2009^a^	Systematic review: 29 articles reviewed to determine if CME leads to an increase in physician knowledge	Physicians and health professionals	Knowledge	Despite low quality of evidence presented in the literature, there is sufficient evidence to confirm an increase in physician knowledge with the use of multimedia, multiple instructional techniques and multiple exposures in CME.
			Technique: multiple	
			Media: multiple	
			Frequency: both single and multiple	
Davis D and Galbraith R 2009^a^	Systematic review: 105 studies reviewed to determine impact of CME on practice behaviours	Health professionals	Practice behaviours	Sufficient evidence to support the use of single, live or multimedia and multiple educational techniques as effective CME methods in changing physician performance. Recommend multiple exposures over single exposures.
			Technique: multiple	
			Media: live	
			Frequency: both single and multiple	
Forsetlund L et al. 2009	Systematic review: 81 articles reviewed to determine the effect of educational meetings on practice behaviours and clinical practice outcomes	More than 11,000 health professionals	Practice behaviours and clinical practice outcomes	Sufficient evidence to conclude that educational meetings alone or combined with other interventions can have a small improvement on professional practice and health care outcomes, but no effect on changing complex behaviours. Previous reviews found that interactive workshops resulted in moderate improvements, whereas didactic sessions did not.
			Technique: multiple	
			Media: live	
			Frequency: single	
Gysels M et al. 2005	Systematic review: 16 articles reviewed to evaluate effective educational techniques for teaching communication skills	Health professionals	Communication skills	Sufficient evidence to recommend communication training programmes that are learner-centred, carried out over a long period of time, and combine didactic theoretical components with practical rehearsal and constructive feedback.
			Technique: multiple	
			Media: multiple	
			Frequency: both single and multiple	
Hamilton R 2005	Systematic review: 24 articles reviewed to determine how to enhance retention of knowledge and skills during and after resuscitation training	Health professionals	Knowledge, skills	Sufficient evidence to recommend in-hospital simulation to teach resuscitation training for nurses in clinical areas in addition to remedial training and the availability of resuscitation equipment for self-study. Video self-instruction has been shown to improve competence in resuscitation.
			Technique: multiple	
			Media: multiple	
			Frequency: both single and multiple	
Marinopoulos S et al. 2007^a^	Systematic review: from 68,000 citations, 136 studies and nine systematic reviews were identified and reviewed	Health professionals and allied health professionals	Knowledge, skills, practice behaviours and clinical practice outcomes	Firm conclusions are not possible due to the overall low quality of the literature. Despite this, the literature supported the concept that CME was effective at the acquisition and retention of knowledge, attitudes, skills, behaviours and clinical outcomes. Common themes included that live media was more effective than print, multimedia was more effective than single media interventions, multiple exposures were more effective than a single exposure, and simulation methods are effective in the dissemination of psychomotor and procedural skills.
			Technique: multiple	
			Media: live	
			Frequency: both single and multiple	
Mansouri M and Lockyer J 2007	Meta-analysis: 31 studies reviewed to determine the impact of CME on knowledge, skills and clinical practice outcomes	Mostly physicians	Knowledge, skills, practice behaviours and clinical practice outcomes	Sufficient information to conclude that the impact of CME on physician performance and patient outcome is small, but has a medium effect on knowledge and a larger effect when the interventions are interactive, use multiple methods and are designed for a small group of physicians from a single discipline.
			Technique: multiple	
			Media: live	
			Frequency: both single and multiple	
Mazmanian P et al. 2009^a^	Systematic review: 37 articles reviewed to determine the impact of CME on clinical practice outcomes	Physicians, nurse-practitioners, nurses, allied health professionals	Clinical practice outcomes	Due to low quality of evidence, there is no firm conclusion on the impact of CME on clinical practice outcomes; however, multimedia, multiple techniques of instruction and multiple exposures to content are suggested to meet instructional objectives intended to improve clinical outcomes.
			Technique: multiple	
			Media: multiple	
			Frequency: both single and multiple	
Moores L et al. 2009^a^	Systematic review: 136 articles and nine systematic reviews were reviewed to evaluate what makes CME effective	Health professionals	General	Significant evidence to support the use of CME interventions that use multimedia in instruction, multiple instruction techniques and frequency of exposure, to have a positive effect on knowledge, psychomotor skills, practice performance and clinical outcomes. The use of print media alone is not recommended.
			Technique: multiple	
			Media: multiple	
			Frequency: both single and multiple	
Nestel et al. 2011	Systematic review: 81 articles retrieved to summarize the best evidence related to use of simulation for learning	Health professionals	Psychomotor skills	Sufficient evidence is available to conclude that use of simulation leads to improved knowledge and skill. Studies with low-quality evidence suggest a transfer of skills to the clinical setting. Instructional design and educational theory, contextualization, transferability, accessibility and scalability must all be considered in simulation-based education programmes.
			Technique: multiple	
			Media: multiple	
			Frequency: both single and multiple	
O’Neil K et al. 2009^a^	Systematic review: from the 136 studies identified in the systematic review, 15 articles, 12 addressing physician application of knowledge and three addressing psychomotor skills, were identified and reviewed	Health professionals and allied health professionals	Knowledge, psychomotor skills	Sufficient evidence to support CME as effective in improving physician application of knowledge. Multiple exposures and longer durations of CME are recommended to optimize educational outcomes. Quality of evidence does not address to psychomotor skill development.
			Technique: multiple	
			Media: multiple	
			Frequency: both single and multiple	
Rampatige R et al. 2009	Systematic review: 476 articles selected for inclusion. Section A relates to CPE in general (sections B and C are not relevant); nine studies were reviewed to determine effect of CME on practice behaviours and clinical practice outcomes	Health professionals	Practice behaviours and clinical practice outcomes	Interactive and practice enabling strategies are more useful than print-based and educational meetings. Multiple education efforts combined with good feedback/interaction between educators and learners are most effective. Opinion leaders and outreach visits shown to be effective.
			Technique: multiple	
			Media: multiple	
			Frequency: both single and multiple	
Raza A et al. 2009 (follow-up study to Coomarasamy A and Khan KS 2004)	Systematic review: Cochrane review of 36 studies reviewed to determine evidence to support effective CME	Health professionals	General	Evidence from 16 randomized trials support interactive and clinically integrated learning sessions and interactive classroom teaching as second choice for an effective form of CME. Review demonstrated that interactive workshops improved knowledge and practice behaviours.
			Technique: multiple	
			Media: multiple	
			Frequency: both single and multiple	
Satterlee W et al. 2008	Systematic review: nine articles reviewed to determine impact of CME on clinical practice outcomes	Health professionals	Clinical practice outcomes	Combined didactic presentations and interactive workshops and combined didactic presentations were more effective than traditional didactic presentations alone. The use of multiple interventions over an extended period increased effectiveness. Targeted education should focus on changing a behaviour that is simple, since effect size is inversely proportional to the complexity of the behaviour.
			Technique: multiple	
			Media: multiple	
			Frequency: both single and multiple	
Thomson O’Brien MA et al. 2001	Systematic review: 32 articles reviewed to determine the effect of educational meetings on practice behaviours and clinical practice outcomes	Health professionals	Practice behaviours and clinical practice outcomes	Moderate data quality suggests interactive workshops can result in moderately large changes in professional practice. Didactic sessions alone are unlikely to change professional practice.
		n = 2,995	Technique: multiple	
			Media: live	
			Frequency: single	
Williams J et al. 2008	Systematic review: nine studies reviewed to evaluate if disaster training improves knowledge and skills	Health professionals and allied health professionals	Knowledge, skills	Insufficient data quality exists to report on the impact of disaster response training interventions on knowledge and skills. Data suggest that both computer-based and live instruction may increase knowledge.
			Technique: multiple	
			Media: multiple	
			Frequency: both single and multiple	

^a^JHU EPC systematic review. *CME* Continuing medical education, *JHU EPC* Johns Hopkins University Evidence-Based Practice Center.

The weight of the evidence across several studies indicated that CPE could effectively address knowledge outcomes, although several studies used weaker methodological approaches. Specifically, computer-based instruction was found to be equally or more effective than live instruction for addressing knowledge, while multiple repetitive exposures leads to better knowledge gains than a single exposure. Games can also contribute to knowledge if designed as interactive learning experiences that stimulate higher thinking through analysis, synthesis or evaluation.

No studies or systematic reviews looked only at attitudes, but CPE that includes clinical integration, simulations and feedback may help address attitudes. The JHU EPC group systematic review evaluation of the short- and long-term effects of CPE on physician attitudes reviewed 26 studies and, despite the heterogeneity of the studies, identified trends supporting the use of multimedia and multiple exposures for addressing attitudes [6].

Several systematic reviews looked specifically at skills, concluding that there is weak but sufficient evidence to suggest that psychomotor skills can be addressed with CPE interventions that include simulations, practice with feedback and/or clinical integration. 'Dose-response’ or providing sufficient practice and feedback was identified as important for skill-related outcomes. Other RCTs suggest clinically integrated education for supporting skill development. Choa et al. found that neither the audio mentoring via mobile nor animated graphics via mobile resulted in the desired psychomotor skills, reinforcing the need for practice and feedback for psychomotor skill development identified in other studies [49].

Two systematic reviews focused on communication skills and found techniques that include behaviour modeling, practice and feedback, longer duration or more practice opportunities were more effective [52,53]. Evidence suggests that development of communication skills requires interactive techniques that include practice-oriented strategies and feedback, and limit lecture and print-based materials to supportive strategies only.

Findings also suggest that simulation, PBL, multiple exposures and clinically integrated CPE can improve critical thinking skills. Mobile-based POC support was found to be more useful in the development of critical thinking than print-based job aids.

Several systematic reviews specifically looked at CPE, practice behaviours and the behaviours of the provider. These studies found, despite reportedly weak evidence, that interactive techniques that involved feedback, interaction with the educator, longer durations, multiple exposures, multimedia, multiple techniques and reminders may influence practice behaviours.

A targeted review of 37 articles from the JHU EPC review on the impact of CPE on clinical practice outcomes drew no firm conclusions, but multiple exposures, multimedia and multiple techniques were recommended to improve potential outcomes [6]. Interaction and feedback were found to be more useful than print or educational meetings (systematic review of nine articles) [24], but print-based unsolicited materials were not found to be effective [50]. The systematic review of live, classroom-based, multi-professional training conducted by Rabal et al. found 'the impact on clinical outcomes is limited’ [54].

## Discussion

The heterogeneity of study designs included in this review limits the interpretations that can be drawn. However, there is remarkable similarity between the information from studies included in this review and similar discussions published in the educational psychology literature. We believe that there is sufficient evidence to support efforts to implement and evaluate the combinations of training techniques, frequency, settings and media included in this discussion.

Avoid educational techniques that provide a passive transfer of information, such as lecture and reading, and select techniques that engage the learner in mental processing, for example, case studies, simulation and other interactive strategies. This recommendation is reinforced in educational psychology literature [55]. There is sufficient evidence to endorse the use of simulation as a preferred educational technique, notably for psychomotor, communication or critical thinking skills. Given the lack of evidence for didactic methods, selecting interactive, effective educational techniques remains the critical point to consider when designing CPE interventions.

Self-directed learning was also found to be an effective strategy, but requires the use of interactive techniques that engage the learner. Self-directed learning has the additional advantage of allowing learners to study at their own pace, select times convenient for them and tailor learning to their specific needs.

Limited evidence was found to support team-based learning or the provision of training in work teams. There is a need for further study in this area, given the value of engaging teams that are in the same place at the same time in an in-service training intervention. This finding is especially relevant for emergency skills that require the collaboration and cooperation of a team.

Repetitive exposure is supported in the literature. When possible, replace single-event frequency with targeted, repetitive training that provides reinforcement of important messages, opportunities to practice skills and mechanisms for fostering interaction. Recommendations drawn from the educational psychology literature that address the issue of cognitive overload [56] suggest targeting information to essentials and repetition.

Select the setting based on its ability to deliver effective educational techniques, be similar to the work environment and allow for practice and feedback. In this time of crisis, workplace learning that reduces absenteeism and supports individualized learning is critical. Conclusions from literature in educational psychology reinforce the importance of 'situating’ learning to make the experience as similar to the workplace as possible [57].

Certain common themes emerged from the many articles that commented on the role of media in CPE effectiveness. A number of systematic reviews suggest the use of multimedia in CPE. It is important to note that the studies that found similar knowledge outcomes between computer-based and live instruction stated that both utilized interactive techniques, possibly indicating the effectiveness was due to the technique rather than the media through which it was delivered. While the data on use of mobile technology to deliver CPE were limited, the study by Zurovac et al. indicated the potential power of mobile technology to improve provider adherence to clinical protocols [29]. Currently, there is unprecedented access to basic mobile technology and increasing access to lower-cost tablets and computers. The use of these devices to deliver effective techniques warrants exploration and evaluation, particularly in low- and middle-income countries.

CPE can positively impact desired learning outcomes if effective techniques are used. There are, however, very limited and weak data that directly link CPE to improved clinical practice outcomes. There are also limited data that link CPE to improved clinical practice behaviours, which may influence the strength of the linkage to outcomes.

### Limitations

The following limitations apply to the methodology that we selected for this study. An integrative review of the literature was selected because the majority of published studies of education and training in low- and middle-resource countries did not meet the parameters required of a more rigorous systematic review or meta-analysis. The major limitation of integrative reviews is the potential for bias from their inclusion of non-peer-reviewed information or lower-quality studies. The inclusion of articles representing a range of rigor in their research design restricts the degree of confidence that can be placed on interpretations drawn by the authors of those articles, with the exception of original articles that explicitly discussed quality (such as systematic reviews). This review did not make an additional attempt to reanalyse or combine primary data.

Therefore, for purpose of this article, we also graded all articles and included only tier 1 articles in the analysis. This resulted in restriction of information on certain topics for this report, although a wider range of information is available.

We faced an additional limitation in that many articles included in the review were neither fully transparent nor consistent with terminology definitions used in other reports. This is due in part to the fact that we went beyond the bio-medical literature, to include studies conducted in the education and educational psychology literature, as was appropriate to the integrative review methodology. Certain topics were underdeveloped in the literature, which limits the interpretation that can be drawn on these topics. Other topics are addressed in studies conducted using lower-tier research methodologies (for example observational and/or qualitative studies) that were not included in this article. In addition, the overwhelming majority of studies focused on health professionals in developed or middle-income countries. There were very few articles of sufficient rigor conducted in low- and middle-income countries. This limits what we can say regarding the application of these findings among health workers of a lower educational level and in lower-resourced communities.

## Conclusions

In-service training has been and will remain a significant investment in developing and maintaining essential competencies required for optimal public health in all global service settings. Regrettably, in spite of major investments, we have limited evidence about the effectiveness of the techniques commonly applied across countries, regardless of level of resource.

Nevertheless, all in-service training, wherever delivered, must be evidence-based. As stated in Bloom’s systematic review, 'Didactic techniques and providing printed materials alone clustered in the range of no to low effects, whereas all interactive programmes exhibited mostly moderate to high beneficial effect. … The most commonly used techniques, thus, generally were found to have the least benefit’ [14]. The profusion of mobile technology and increased access to technology present an opportunity to deliver in-service training in many new ways. Given current gaps in high-quality evidence from low- and middle-income countries, the future educational research agenda must include well-constructed evaluations of effective, cost-effective and culturally appropriate combinations of technique, setting, frequency and media, developed for and tested among all levels of health workers in low- and middle-income countries.

## Abbreviations

BEME: Best Evidence in Medical Education; CI: Confidence interval; CINAHL: Cumulative Index to Nursing and Allied Health Literature; CME: Continuing medical education; CPE: Continuing professional education; CPR: Cardiopulmonary resuscitation; EBM: Evidence-based medicine; JHU EPC: Johns Hopkins University Evidence-Based Practice Center; MeSH: Medical subject headings; OCEMB: Oxford Centre for Evidence-Based Medicine; PBL: Problem-based learning; POC: Point-of-care; RCT: Randomized controlled trial.

## Competing interests

The authors declare they have no competing interests.

## Authors’ contributions

JB performed article reviews for inclusion, synthesized data and served as primary author of the analysis and manuscript. PJ conceived the study, participated in its design and coordination, and provided significant input into the manuscript. JF provided guidance on the literature review process, grading and categorizing criteria, and quality review of selected articles, and participated actively as an author of the manuscript. CC and JBT contributed to writing of the manuscript. JA searched the literature, performed initial review and coding, and contributed to selected sections of the manuscript. All authors read and approved the final manuscript.
